# Pretreatment of rice straw with combined process using dilute sulfuric acid and aqueous ammonia

**DOI:** 10.1186/1754-6834-6-109

**Published:** 2013-07-30

**Authors:** Sung Bong Kim, Sang Jun Lee, Ju Hun Lee, You Ree Jung, Laxmi Prasad Thapa, Jun Seok Kim, Youngsoon Um, Chulhwan Park, Seung Wook Kim

**Affiliations:** 1 Department of Chemical and Biological Engineering, Korea University, Seoul, 136-701, South Korea; 2 Department of Chemical Engineering, Kyonggi University, Suwon 443-760, South Korea; 3 Clean Energy Research Center, Korea Institute of Science and Technology, Seoul 136-791, South Korea; 4 Department of Chemical Engineering, Kwangwoon University, Seoul 139-701, South Korea

**Keywords:** Rice straw, Pretreatment, Soaking aqueous ammonia, Dilute acid pretreatment, Response surface methodology

## Abstract

**Background:**

Use of lignocellulosic biomass has received attention lately because it can be converted into various versatile chemical compounds by biological processes. In this study, a two-step pretreatment with dilute sulfuric acid and aqueous ammonia was performed efficiently on rice straw to obtain fermentable sugar. The soaking in aqueous ammonia process was also optimized by a statistical method.

**Results:**

Response surface methodology was employed. The determination coefficient (R^2^) value was found to be 0.9607 and the coefficient of variance was 6.77. The optimal pretreatment conditions were a temperature of 42.75°C, an aqueous ammonia concentration of 20.93%, and a reaction time of 48 h. The optimal enzyme concentration for saccharification was 30 filter paper units. The crystallinity index was approximately 60.23% and the Fourier transform infrared results showed the distinct peaks of glucan. Ethanol production using *Saccharomyces cerevisiae* K35 was performed to verify whether the glucose saccharified from rice straw was fermentable.

**Conclusions:**

The combined pretreatment using dilute sulfuric acid and aqueous ammonia on rice straw efficiently yielded fermentable sugar and achieved almost the same crystallinity index as that of α-cellulose.

## Background

Biomass pretreatments are key steps in the low-cost bioconversion of cellulosic biomass to sugar because of the rigid and hard-to-degrade structure of the biomass cell walls. Pretreatments are used to release cellulose from amorphous lignin and hemicellulose. Chemical pretreatments using acid and alkali reagents have been widely studied because of their simplicity and efficient performance.

Acid pretreatments hydrolyze plant cell walls, especially their hemicellulose component. H_2_SO_4_, HNO_3_, and HCl are usually used for acid pretreatments in dilute and acidic states [1-4]. The solubilized hemicellulose can be converted to xylose, a monomer, in acidic media, and the xylose can then be overdegraded in a strongly acidic environment [5,6]. Though glucose and xylose can biologically yield versatile building block products of various biochemicals, they can also be overdegraded and converted to by-products such as furfural and hydroxymethylfurfural (HMF), respectively [3,7]. Therefore, in order to achieve selective hydrolysis using an acid reagent, an appropriate acid concentration, reaction temperature, and other critical factors must be experimentally determined.

Alkaline pretreatments have also been extensively studied for modifying cell walls. During such pretreatments, solvation and saponification reactions take place [3]. As a result, the biomass swells, and access to its inner space by saccharification enzymes is enhanced [3,8]. Also, alkali pretreatments selectively remove lignin portion mainly. Xylan and lignin support the cellulose backbone that includes the biomass cell wall [1,3]. Though ammonia has been widely used in alkali pretreatments, its use leads to many environmental problems because of which recovery and recycling processes must also be used along with this pretreatment. NaOH and KOH have also been evaluated for use in alkali pretreatments because they are cheaper than ammonia [9,10].

In our previous work, rice straw was pretreated with dilute sulfuric acid and pretreatment using the compounds was analyzed. In addition, to avoid the overdegradation of biomass, the pretreatment was optimized using a statistical model and a computer program [11]. As xylose is obtained from hemicellulose, lignin can also be converted into useful compounds such as organic solvents, aromatic compounds, and fuel additives by chemical and biological methods. Removal of hemicellulose and lignin could dramatically improve enzyme digestibility by enhancing the enzyme accessibility to cellulose. The pretreatment with dilute sulfuric acid followed by aqueous ammonia would then be needed for efficient saccharification and lignin isolation.

**Figure 1:**
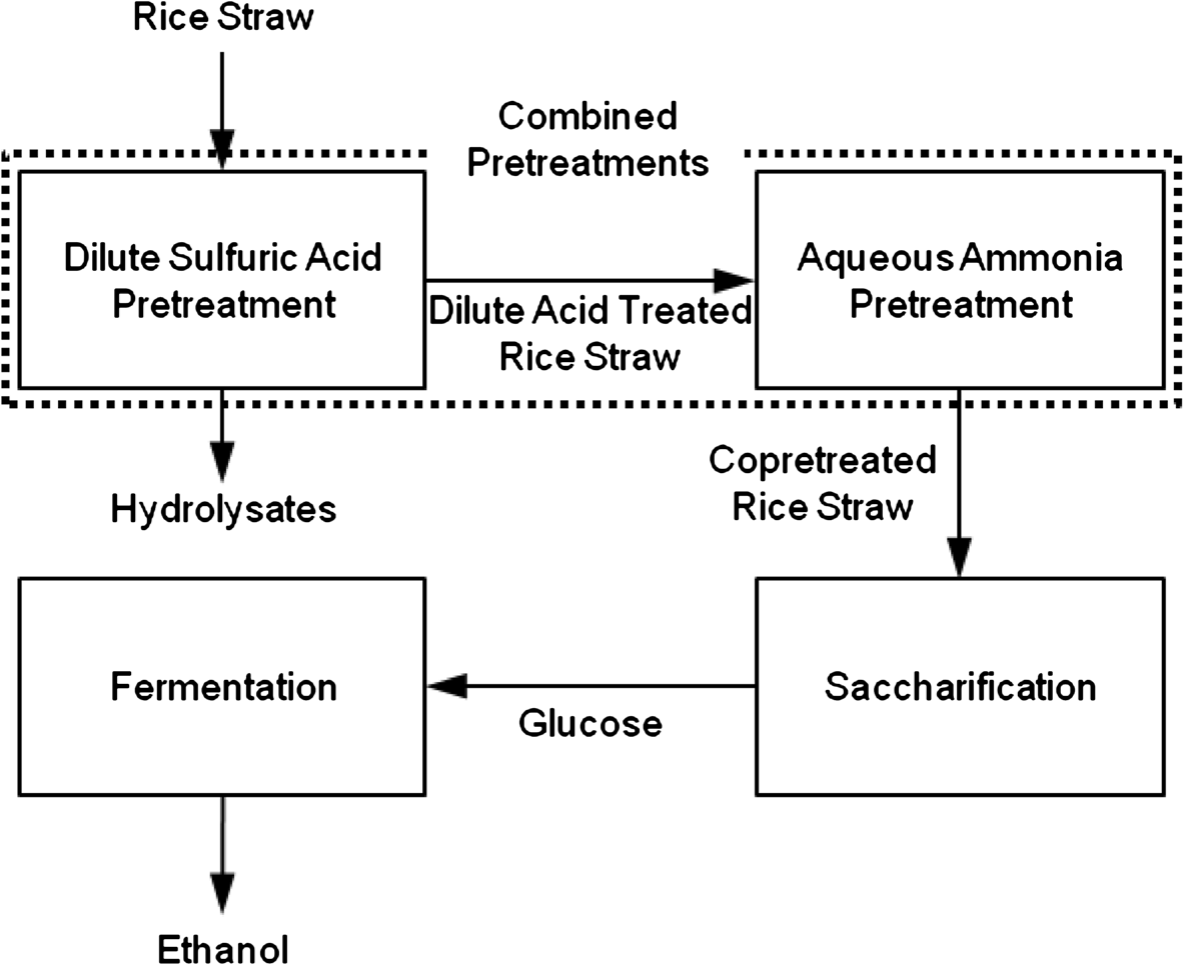
Schematic depiction of processes used in this study.

In this study, rice straw was pretreated with dilute sulfuric acid under the conditions previously described [11]. The rice straw that had been subjected to dilute-acid pretreatments to remove a large portion of amorphous hemicellulose was pretreated again with aqueous ammonia to remove lignin. The process of soaking in aqueous ammonia (SAA) was optimized, and pretreatment factors were analyzed using a statistical method and model. After statistical analysis, X-ray diffractometer (XRD) was utilized to measure the crystallinity index (CrI), and Fourier transform infrared (FTIR) analysis was performed to investigate the molecule structure of rice straw. The rice straw, which was copretreated with dilute acid and aqueous ammonia, was saccharified using saccharification enzymes. Figure 1 shows the schematic depiction of the processes used in this study.

## Results and discussion

### Response surface methodology analysis

Rice straw was pretreated with dilute sulfuric acid under previously optimized conditions: 142°C temperature, 1.21% concentration of sulfuric acid, and 11.6 min reaction time [11]. After the pretreatment, the solid portion comprising the treated rice straw and the liquid portion of hydrolysates containing the solubilized xylose was separated by filtration. The solids were washed with distilled water and subsequently treated with aqueous ammonia. The pretreatment with aqueous ammonia was optimized using a statistical method.

The factors affecting the statistical analysis were temperature, concentration of aqueous ammonia, and reaction time that were determined from fundamental experiments. In the pretreatment process with aqueous ammonia, the aforementioned three factors strongly and directly affected either the productivity of sugar or the energy cost of the process. Fundamental experiments to determine the range of each of these factors were performed before the experiment using a statistical model. The temperature range, aqueous ammonia concentration, and reaction time were employed as described in the “Methods” section. The amount of glucose recovered from saccharification after SAA for each designed condition was used as the data for the statistical analysis.

Central composite design (CCD) was used to investigate the effects of different factors on the rice straw pretreatment. Design Expert® 6.0 software was employed to obtain the analysis of variance (ANOVA), regression coefficients, and polynomial regression equation from the data of the CCD experiment. Table 1 shows the results of ANOVA of the CCD model.

**Table 1:** Results of analysis of variance (ANOVA)

**Source**	**DF**	**Sum of squares**	**Mean square**	**F value**	**Pr > F**
**Model**	9	5103.98	567.11	27.15	<.0001
**Error**	10	208.278	20.82		
**Corrected total**	19	5314.738			

CV = 6.77, R^2^ = 0.9607.

**Figure 2:**
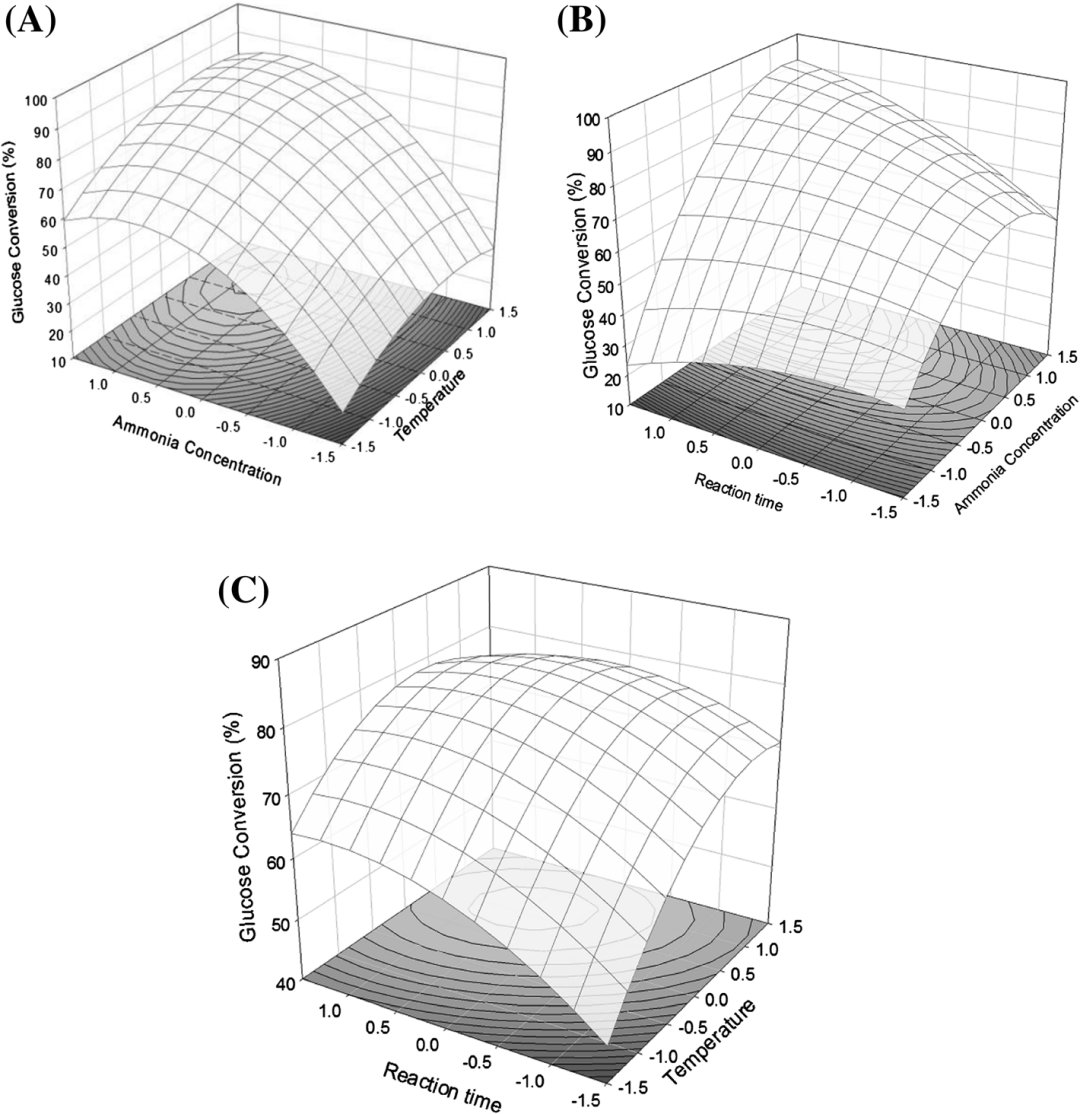
**Response surface plots of effect of each factor on glucose yield from soaking in aqueous ammonia process. (A)**: Effects of temperature and aqueous ammonia concentration; **(B)**: Effects of aqueous ammonia concentration and reaction time; **(C)**: Effects of temperature and reaction time.

The coefficient of variation (CV) is the degree of precision with which experiments are similar. In the current study, a CV of 6.77% was obtained. The CV is a function of the standard deviation and standardizes the scale of data. In general, a lower CV indicates that the factors are highly reliable during the optimization. The determination coefficient (R^2^) was 0.9607 (96.07%), and a higher determination coefficient indicated a higher reliability regarding the relationship between the observed experimental data and designed predictions. A high F-value of 27.15, indicating a high efficiency of the factors, was obtained (Table 1). Furthermore, P-value, which is important to understanding the pattern of mutual interactions between the variables, was lower than 0.0001 (Table 1). Usually, when P-value is lower than 0.05, the factors are considerably varied and statistically significant. Table 2 shows the statistical analysis of the factors. The following polynomial equation was obtained using multiple regression analysis. The most effective factor was ammonia concentration, which had an F-value of 136.73; the temperature was also found to be critical.

$$\begin{array}{l} Y=78.14-5.81X_{1}+14.46X_{2}\\ \;+3.51X_{3}-3.96X_{11}-9.06X_{22}-2.50X_{33}\\ \;+1.20X_{12}-1.65X_{13}+5.48X_{23} \end{array}$$

where *X*_*1*_ is a coded value of the temperature, *X*_*2*_ is a coded value of the ammonia concentration, and *X*_*3*_ is a coded value of the reaction time. Once the derived equation (PDE) was solved, three contour plots and a three-dimensional mesh were obtained (Figure 2). Figure 2(A) is a plot of *X*_*1*_ vs. *X*_*2*_, Figure 2(B) depicts *X*_*2*_ vs. *X*_*3*_, and Figure 2(C) is a plot of *X*_*1*_ vs. *X*_*3*_. The graphs of three-dimensional mesh with each contour plot show the effect of each factor and the approximate optimal point. The shapes of the response surfaces indicated the nature and extent of the interactions between the different factors [12,13]. In nature, a high temperature and a long reaction time might not decrease the enzyme digestibility. However, the three-dimensional shape decreased when these aforementioned factors increased, which affected the regression of the design of experiment (DOE) results. However, the regression surfaces indicated that as these factors increased, conversion of glucose increased, and that the axis of the reaction time factor was not much different than that of the other factors. In addition, at the appropriate aqueous ammonia concentration and temperature, the effect of the reaction time was not significant after 12 h. Also, the perturbation in the conversions of *X*_*2*_ was larger than that of others, and the difference of *X*_*2*_ in range was based on the F-values.

**Table 2:** Statistical analysis of factors

**Source**	**DF**	**Mean square**	**F value**	**Pr > F**
**X**_**1**_	1	460.91	22.07	0.0008
**X**_**2**_	1	2855.64	136.74	<.0001
**X**_**3**_	1	169.05	8.06	0.0176
**X**_**11**_	1	124.56	10.82	0.0082
**X**_**22**_	1	1130.13	56.67	<.0001
**X**_**33**_	1	90.10	4.32	0.0644
**X**_**12**_	1	11.23	0.55	0.4761
**X**_**13**_	1	22.11	1.04	0.3309
**X**_**23**_	1	2241.12	11.50	0.0069

X_1_: Temperature.

X_2_: Concentration of aqueous ammonia.

X_3_: Reaction time.

### Optimization, confirmation, and enzyme loading test

The optimal coded values, determined by response surface methodology (RSM), were −0.8625 for temperature (*X*_*1*_), 0.7412 for aqueous ammonia concentration (*X*_*2*_), and 0 for reaction time (*X*_*3*_), whereas the real values were 48.75°C, 20.93%, and 48 h, respectively. In the statistical analysis and optimization, wherein the results were analyzed based on the numerical regression level, the optimal reaction time was approximately 79 h. Though the process lasted for over 80 h, the yield was not notable compared to that obtained with a reaction time of 48 h.

Some portion of lignin was removed and the biomass structure was swollen; however, maximum glucose recovery or initial reaction rate could not be enhanced. In addition, there was no significant improvement in the initial reaction rate and maximum glucose conversion even when more than 50% of the lignin was solubilized. Reaction time factors were closely associated with the energy cost, and the SAA was carried out for 24–48 h. Design Expert® provided the adjustment of the optimal result so as to be suitable for the real process, and the reaction time was limited to less than the reaction time of 48 h. Thus, the analysis conditions were minimized with regard to temperature and 48 h over a forced maximum range, and optimum of 48 h were derived by the function of statistical program. After the pretreatment with the derived optimum conditions, solid analysis of rice straw was performed to confirm the change in the rice straw composition of each sample. All experiments were conducted in triplicate. Table 3 lists the results of the comparison of the compositions of raw rice straw (RR), dilute-acid-pretreated rice straw (DR), and dilute acid and aqueous ammonia combined pretreated rice straw (DAR). The RR had approximately 39.34% glucan. The glucan portion was, in practice, the quantity of theoretical maximum. In the case of DR, the glucan portion increased to 68.38% because most of XMG (xylan, mannan and galactan) was removed. When a SAA pretreatment was performed on the DR, approximately 79.75% glucan was observed because of the removal of xylan and lignin, two major structural inhibitors. The determined theoretical maximum quantity of glucose for RR was 7.86 g/L, for DR was 13.67 g/L, and for DAR was 15.95 g/L. The derived optima were confirmed by saccharification, and when these conditions were used for the pretreatment, 78.04% (approximately 12.44 g/L) of glucose was obtained, similar to those observed with a reaction time of 79 h (78.42%, 12.50 g/L). This similarity was expected because of the low F-value of the reaction time factor in the statistical analysis. The predicted maximum value for the saccharified glucose was approximately 75.16% [14].

**Table 3:** Effect of pretreatments on rice straw composition

	**Raw rice straw (RR)**	**Dilute acid pretreated rice straw (DR)**	**Combined pretreated rice straw (DAR)**
**Glucan**	39.34 ± 2.35% (TM = 7.86 g/L)	68.38 ± 1.71% (TM = 13.67 g/L)	79.75 ± 3.87% (TM = 15.95 g/L)
**XMG**	28.46 ± 2.07%	10.15 ± 1.05%	10.87 ± 1.56%
**Others**	30.2 ± 1.68%	21.47 ± 1.52%	9.38 ± 1.01%

※XMG: xylan, mannan and galactan.

※Others: containing lignin and ash.

※TM: theoretical maximum.

**Figure 3:**
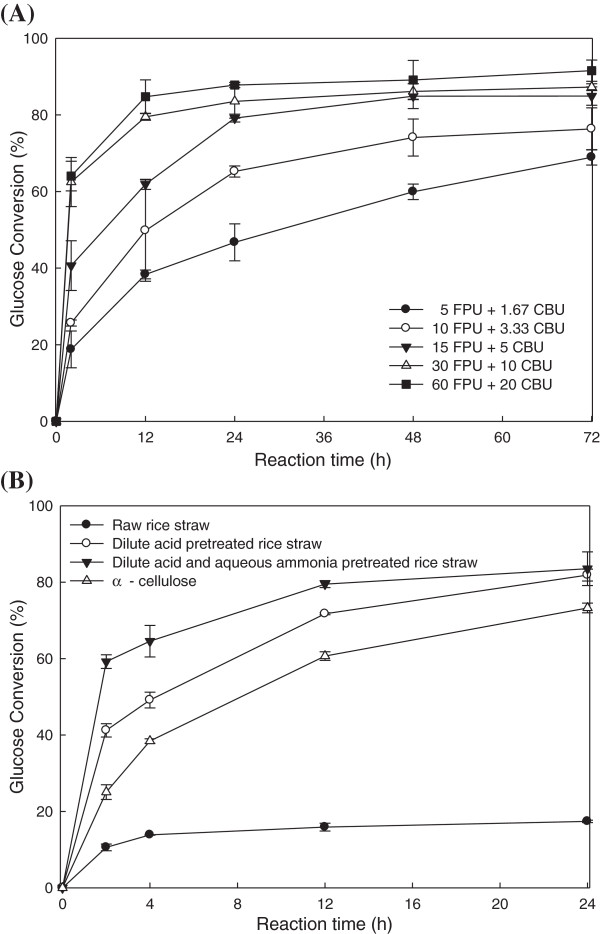
**Saccharification of pretreated and untreated rice straw. (A)**: Enzyme loading test of DAR; **(B)**: Comparison of RR, DR, DAR, and α-cellulose in enzyme media of 30 FPU Celluclast 1.5 L and 10 CBU Novozyme 188.

After two steps of the pretreatment with dilute acid and aqueous ammonia, saccharification with enzyme loading test was performed on 5–60 filter paper units (FPU) of Celluclast (cellulase) and 1.7–20 cellobiase units (CBU) of Novozyme 188 (β-glucosidase). Figure 3(A) shows the results of enzyme-loading test. When 30 FPU Cellulase and 10 CBU β-glucosidase were utilized for saccharification, the value of glucose conversion obtained was approximately 80.12% of the theoretical maximum at 12 h. After 72 h reaction time, 86.43% glucose was recovered, and the initial reaction rate (at 2 h) was 1.384 × 10^-3^ g/L · s. Approximately 84.71% glucose was obtained after 12 h when 60 FPU with 20 CBU were used, and the initial reaction rate was 1.418 × 10^-3^ g/L · s. The maximum conversion after 72 h and the initial reaction rate (at 2 h) were similar at 30 FPU and 60 FPU, but the conversion of glucose was not notable with regard to the maximum or initial reaction rate below 30 FPU. Thus, 30 FPU with 10 CBU was determined to be the appropriate enzyme-loading dosage.

Figure 3(B) shows the comparison of the RR, DR and DAR in saccharification using 30 FPU of Celluclast and 10 CBU of Novozyme 188. When dilute sulfuric acid was employed, initial reaction rate was 7.657 × 10^-4^ g/L · s, which was 6.61 times higher than that of the RR (1.158 × 10^-4^ g/L · s). When pretreatments with dilute sulfuric acid and aqueous ammonia were employed, the initial reaction rate was approximately 11.94-fold higher (1.384 × 10^-3^ g/L · s) than the values obtained for RR. Thus, pretreatments with dilute acid and aqueous ammonia were more effective compared to a one-step pretreatment. Ko *et al.* treated rice straw using aqueous ammonia and obtained 71.1% glucose conversion [15]. Chen *et al.* suggested the use of a dilute acid and steam explosion to treat rice straw, and as a result, glucose conversion of approximately 85% was obtained [16]. The results of the current study were notable compared to the earlier results. Also, Kim *et al.* reported a two-stage pretreatment of rice straw using aqueous ammonia and dilute acid in a facilitating apparatus and obtained approximately 89% glucose conversion [17]. Our study employed SAA under atmospheric conditions and yielded results that were significant within the statistical error range.

### Analysis of pretreated biomass

Chemically treated rice straw was analyzed by XRD and FTIR to investigate the change in rice straw before and after the pretreatment.

**Figure 4:**
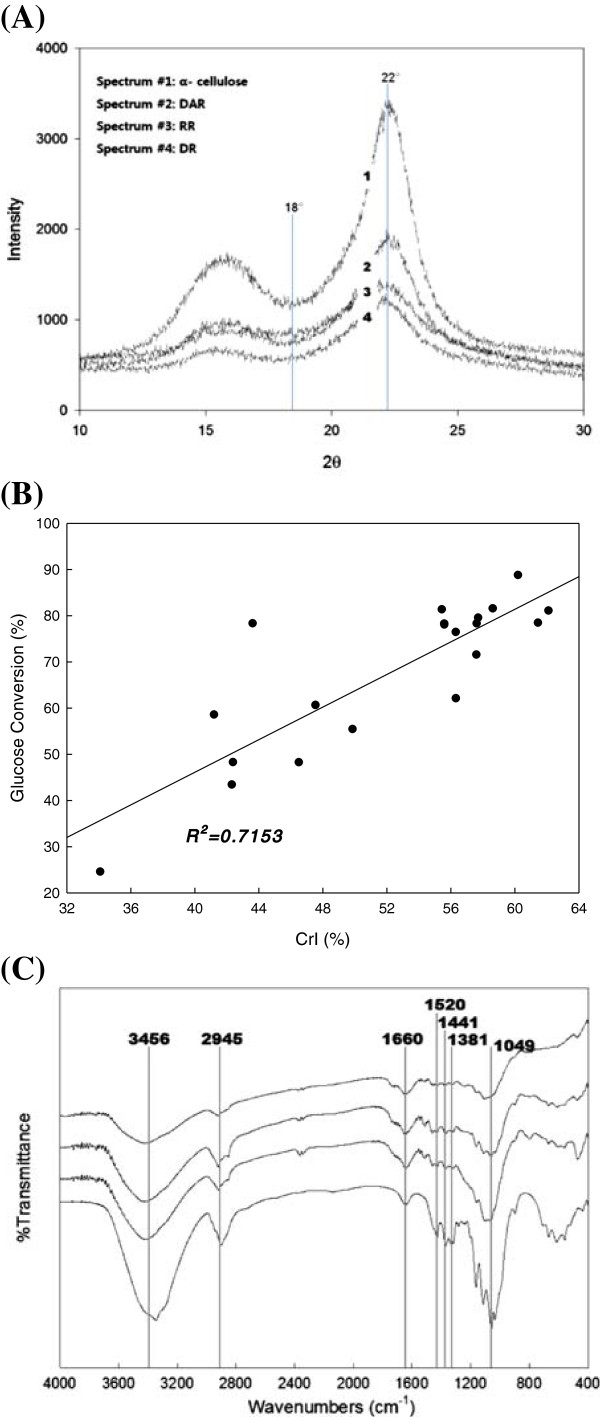
**Instrumental analysis of biomass before and after pretreatment. (A)**: Results of XRD. Crystalline region at 2θ = 22° and amorphous portion at 2θ = 18° were used to calculate crystallinity index (CrI); **(B)**: Relationship of CrI with enzyme digestibility; **(C)**: Results of FTIR spectroscopy.

The CrI was measured using XRD, where an increase in the CrI represented enhanced enzyme digestibility [8,18,19]. Figure 4(A) shows the results of the XRD analysis. The crystalline portion was 22° and the amorphous portion was 18°. Spectrum-3 corresponded to RR and its CrI was 35.42%. The CrI of DR (spectrum-4) was approximately 46.68%, which was higher than that of the RR. When rice straw was sequentially treated under the optimized conditions of dilute acid and aqueous ammonia (DAR, spectrum-2), CrI increased to 60.23%, which was similar to that of α-cellulose (60.92%, spectrum-1), which is pure cellulose composed of crystalline and amorphous cellulose. In the combined pretreatment with a dilute acid and an alkali, a significant portion of the amorphous material, such as xylan and lignin, was removed. At the same time, either a small portion of the crystalline material, such as glucan, was removed or the crystalline structure was broken by swelling or hydrolysis. Thus, the portion of the exposed crystalline structure could be increased compared to either RR or DR. Although the CrI values of DAR as well as those of α-cellulose were similar, the actual states of the micro structure could be different.

Moreover, correlation of glucose conversion with the CrI values of RR, DR, and DAR were analyzed by the linear regression of the plotted data. Figure 4(B) shows the results of regression between the CrI and glucose conversion. The relation was approximately proportional, which meant that an increase in the CrI corresponded to the removal of barriers for enzymatic accessibility and a relatively high CrI indicated high enzyme digestibility. The value of R^2^ was 0.7153. Many researchers have examined the relationship between the CrI and enzyme digestibility. Fan *et al.* (1987) studied this relationship and reported similar results [19]. Kim *et al.* (2003) studied the effect of ammonia pretreatments on corn stover and found that an increase in CrI roughly corresponded to an increase in enzyme digestibility [20].

Figure 4(C) shows the results of the FTIR analysis that was performed to prove that the crystalline portion was cellulose. Sun *et al.* (2002) performed such analysis and reported that the biomass structure was composed of β-glucosidic bonds and carbon hydrates [21]. The control spectrum is no. 1, which corresponded to α-cellulose. The band intensities provided the following information: 3456 cm^-1^ indicated an O-H group, 2945 cm^-1^ indicated a CH group, 1381 cm^-1^ indicated C-CH_3_ group, 1660 cm^-1^ indicated a β-glucosidic bond between the sugar monomers, and 1520 cm^-1^ and 1441 cm^-1^ indicated an aromatic ring. Spectrum-4 is RR, spectrum-3 is DR, and spectrum-2 is DAR. The peaks in spectrum-4 were blunt and not as sharp as those in spectrum-1, while those in spectrum-3 were less blunt than those in spectrum 4. In contrast, the peaks in spectrum-2 were sharper and clearer. These results indicated that the material became purer after the pretreatment. Thus, these band intensities showed the specific functions and bonds that corresponded to the cellulose structure. By using these pretreatment steps, barriers to enzyme accessibility were removed from the structure of the rice straw, and consequently, the cellulose portion was exposed [20,21].

### Fermentation of glucose

**Figure 5:**
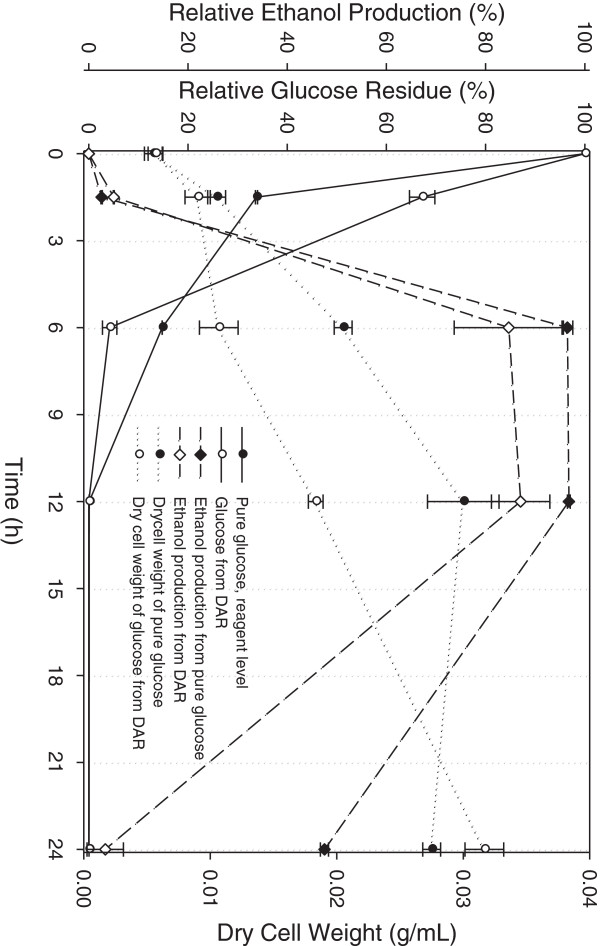
**Results of fermentation of glucose by *Saccharomyces cervisiae *K35.** Fermentation for ethanol production was performed at 30°C for 12 h. Initial concentration of saccharified glucose was approximately 31 g/L.

Fermentation was conducted using glucose produced by saccharification. Then, enzyme deactivation was performed by boiling, followed by centrifugation to remove the solids. The supernatants were collected and concentrated using an evaporator. The glucose concentration was increased to 62 g/L (approx. 6%), which was over four times the concentration of the initial glucose solution recovered from the DAR. The glucose solution was diluted by the same amount of nutrients solution, and the initial glucose concentration before fermentation was approximately 31 g/L. The fermentation was carried out with *Saccharomyces cerevisiae* K35 at 30°C and 200 rpm for 24 h. Control fermentation was performed using pure glucose of reagent quality under the aforementioned conditions of fermentation. The fermentation profiles are shown in Figure 5. Over 90% of glucose was consumed after 6 h, and ethanol production reached approximately 83.25%. The fermentation was independently conducted five times and the deviation was relatively higher than control fermentation, probably because of the effect of inhibitors. This means that the recovered glucose was fermentable but an additional process was needed to remove the inhibitors needed before fermentation. Lee *et al.* have reported the effects of the inhibitory compounds, particularly on the ethanol production [22,23].

Also, trace amounts of the pretreatment reagent could have remained in the fermentation media. Ammonia is a strong and effective reagent for alkali pretreatments, but it is environmentally harmful and could affect the growth of microorganism. Thus, it needs to be recovered and recycled. In practice, SAA is a batch process. The current work deals with the optimization of the pretreatment conditions and could be utilized in processes involving the recycling and percolation of chemical reagents.

## Conclusion

Rice straw was pretreated in a two-step sequential process using dilute sulfuric acid and aqueous ammonia. Statistical studies were performed for the pretreatment process and the results indicated that the model and experiments were reliable and significant. The optimal conditions were found to be a temperature of approximately 42.74°C, ammonia concentration of approximately 20.93%, and a reaction time of 48 h. When the pretreatment was performed using the optimal conditions, approximately 13.91 g/L (approximately 87.24% of the theoretical maximum) of fermentable glucose was recovered. Fermentation process using the recovered glucose yielded ethanol at approximately 83% of the theoretical maximum. The combined pretreatment of rice straw with dilute acid and aqueous ammonia was effective, and this was supported by saccharification, fermentation, XRD, and FTIR analyses.

## Methods

### Feedstock and chemicals

Rice straw was obtained from the Biochemical Engineering Laboratory in Kyonggi University, Suwon, Korea, and stored at 20°C with a relative humidity of 70% in the dark. The rice straw was ground and homogenized using a sieve of 40–60 mesh. Sulfuric acid (H_2_SO_4_) and aqueous ammonia (NH_3_∙H_2_O) were utilized for the pretreatment. Both were purchased from Dea-jung Chemical, Korea.

### Assay method involving enzymes

Celluclast 1.5 L (cellulase) from *Trichoderma reesei* (Novozymes, Denmark) and Novozyme188 (β-glucosidase) from *Aspergillus niger* (Novozymes, Denmark) were used for the enzymatic hydrolysis of biomass. FPU for cellulase and CBU units for β-glucosidase were employed to measure the activity of enzymes. To measure FPU, enzymes were diluted at several levels. Then, 1.0 mL of the diluted Celluclast and 1.0 mL of 0.05 M citrate buffer (pH 4.8) were transferred into test tubes containing 50 mg of filter paper (Whatman No. 1, 1.1 × 6 cm). An assay reaction was performed accurately at 50°C for 60 min. Thereafter, dinitrosalicylic acid (DNS) was added to stop the reaction. The tubes were transferred to a water bath kept at 100°C, and the enzymes were deactivated for 5 min, followed by the addition of 10 mL of distilled water. The colored content of the tube was analyzed using a UV spectrophotometer at 575 nm. The β-glucosidase activity was assayed in 1.0 mL of a reaction mixture containing 0.1 mL of the diluted enzyme solution and 0.9 mL of 1 mM *p*-nitrophenyl-*β*-D-glucopyranoside (*p*NPG) in 0.05 M citrate buffer (pH 4.8) at 50°C for 30 min. Then, 1 M Na_2_CO_3_ solution was added to the mixture and allowed to develop a color. Later, 10 mL of distilled water was added and the release of *p*-nitrophenol was confirmed at 400 nm. The activities of Celluclast and Novozyme188 were 60 FPU and 30 CBU, respectively.

### Pretreatment processes of biomass

The pretreatment of rice straw with dilute sulfuric acid was performed in an oil bath using a well-sealed tube reactor that was 1.2 cm in diameter and 18 cm in length. Preheating, reaction, and cooling were performed in the oil bath. The temperature of the preheating bath was maintained at 210°C for faster heat transfer whereas the cooling bath was kept at room temperature. The temperature, sulfuric acid concentration, and reaction time were similar to those reported in our previous work: 142°C, 1.21% and 11.6 min, respectively [11]. After the pretreatment, solid–liquid separation was conducted, and the solids (rice straw) were extracted, washed, and dried. Later, the pretreatment with aqueous ammonia was carried out at 26.36–93.64°C, ammonia concentrations of 1.54–28.45%, and reaction times of 7.63–88.36 h. The agitation speed and solid–liquid ratio were 250 rpm and 1:12, respectively. This pretreatment step was performed in a 100-mL capped bottle. The ranges of the aforementioned parameters were determined by the fundamental experiments based on other reports [20,24]. After the pretreatment, solids separated by filtration were washed with distilled water to remove the residual ammonia and establish a neutral pH followed by drying at 50°C until the weight became constant [25,26].

### Experimental design and statistical analysis

In the fundamental experiment, temperature, concentration of aqueous ammonia, and reaction time were deemed to be critical factors. The experimental design was constructed using a CCD with α = (2^n^)^1/4^, and Table 4 shows the independent variables as well as the corresponding coded values for RSM. *X*_*1*_*, X*_*2*_ and *X*_*3*_ correspond to temperature, concentration of ammonia, and reaction time, respectively.

**Table 4:** Independent variables and coded values

**Factors**		**−1.682**	**−1**	**0**	**+1**	**+1.682**
**Temperature (°C)**	**(X**_**1**_**)**	26.36	40	60	80	93.64
**Concentration of aqueous ammonia (%, v/v)**	**(X**_**2**_**)**	1.54	7	15	23	28.45
**Reaction time (h)**	**(X**_**3**_**)**	7.63	24	48	72	88.36

The results of 20 experiments were collated to optimize the conditions of alkaline pretreatments with aqueous ammonia. The variables were symbolized as follows:

$$x_{i}=\left(X_{i}–X_{0}\right)/△X\;\left(i=1,2,3,\ldots ,j\right)$$

where *x*_*i*_ is the coded value of the variable *X*_*i*_, *X*_*0*_ is the independent variable real value at the center point, and *△X* is the step change value. The behavior of the system was explained by the following second-degree polynomial equation:

$$y=\beta_{0}+\Sigma \;\beta_{i}X_{i}+\Sigma \;\beta_{\mathrm{ii}}X_{i}{}^{2}+\Sigma \;\beta_{\mathrm{ij}}X_{i}X_{j}$$

where y is the predicted response, X_i_ and X_j_ are the input variables that influence the response variable Y, β_0_ is the offset term, β_i_ is the i^th^ linear coefficient, β_ii_ is the quadratic coefficient, and β_ιj_ is the ij^th^ interaction coefficient. The experimental design and the observed and predicted values of 20 experiments are presented in Table 5. The maximum values of the solubility yield of glucose from saccharification were taken as responses of the designed experiments.

**Table 5:** Design of experiment (DOE), and results of DOE and statistical predictions

**Run**	**X**_**1**_	**X**_**2**_	**X**_**3**_	**Observed glucose (%)**	**Predicted glucose (%)**	**Residual**	**95% confidence limits for mean predicted value**	
**1**	−1	−1	−1	48.17	43.85	4.32	35.53	52.17
**2**	+1	−1	−1	60.55	56.41	4.14	48.09	64.73
**3**	−1	+1	−1	62.01	59.43	2.58	51.11	67.75
**4**	+1	+1	−1	78.22	76.73	1.49	68.41	85.05
**5**	−1	−1	+1	43.32	43.23	0.09	34.91	51.55
**6**	+1	−1	+1	48.14	49.14	−1.00	40.82	57.46
**7**	−1	+1	+1	78.21	80.77	−2.56	72.45	89.09
**8**	+1	+1	+1	88.68	91.42	−2.74	83.10	99.74
**9**	−1.682	0	0	55.32	57.18	−1.86	49.26	65.11
**10**	+1.682	0	0	76.35	76.70	−0.35	68.78	84.63
**11**	0	−1.682	0	24.46	28.18	−3.72	20.25	36.10
**12**	0	+1.682	0	78.35	76.84	1.51	68.92	84.77
**13**	0	0	−1.682	58.47	65.15	−6.68	57.22	73.7
**14**	0	0	+1.682	81.45	76.98	4.47	69.06	84.91
**15**	0	0	0	77.96	78.14	−0.18	73.99	82.29
**16**	0	0	0	79.45	78.14	1.31	73.99	82.29
**17**	0	0	0	80.98	78.14	2.84	73.99	82.29
**18**	0	0	0	81.25	78.14	3.11	73.99	82.29
**19**	0	0	0	78.15	78.14	0.01	73.99	82.29
**20**	0	0	0	71.45	78.14	−6.69	73.99	82.29

X_1_: Temperature.

X_2_: Concentration of aqueous ammonia.

X_3_: Reaction time.

A CCD for three independent variables, each at five levels, was employed to fit a second-order polynomial model, which required 20 experiments [27,28]. The Design-Expert® 6.0 package program (Stat-Ease, USA) was used for experimental design, regression analysis of data, and estimation of the coefficients of the regression equation.

### Saccharification and fermentation

Enzymatic hydrolysis for enzyme digestibility was investigated according to the NREL standard procedure [12]. The reactions were performed at 50°C in 0.05 M of citrate buffer (pH 4.8) at 150 rpm. Fermentation with glucose, produced by saccharification, was performed using *Saccharomyces cerevisiae* K35 [22,29]. The cells were inoculated with the YM broth medium and incubated at 30°C and 200 rpm for 24 h. For the main culture, 0.5 mL of inoculums in the main medium was prepared in 250 mL Erlenmeyer flasks. The main medium was 25 mL of distilled water containing 0.5 g yeast extract, 0.5 g peptone, 0.1 g MgSO_4_∙7H_2_O, and 0.1 g K_2_HPO_4_, to which 25 mL of a saccharified liquid containing 62 g/L glucose was added. The initial pH and temperature of the fermentation process were kept at 5.0 and 30°C, respectively, for 12 h. On the completion of the fermentation process, solid–liquid separation by centrifugation was performed at 8000 rpm for 30 min, and the solids were separated and dried to measure the dry cell weight.

### Analytical methods

Analysis of the solid biomass was performed to determine its absolute composition, according to the standard procedures of the National Renewable Energy Laboratory (NREL, USA) [30]. For acid hydrolysis, biomass was incubated with sulfuric acid (72%, w/w) at 25°C. After the primary hydrolysis, the solution was diluted to 4% and heated to 121°C in an autoclave. On cooling, the mixture was neutralized with calcium carbonate. The supernatant of the biomass composition was then analyzed by high-performance liquid chromatography (HPLC) using an Aminex HPX-87H ion exclusion column (Bio-Rad) and a refractive index detector. The HPLC conditions included a column at 50°C, a mobile phase of 0.005 N H_2_SO_4_, and a flow rate of 0.8 mL/min. The amount of glucose after saccharification and ethanol production yield were also measured by HPLC. All calculations of production and mass balance were performed by considering the biomass composition. This method was based on the solid biomass analysis of NREL [30]. While executing the experimental steps, the biomass was carefully manipulated to prevent weight loss.

To measure the CrI, the XRD (X’pert pro, PANanalytical, the Netherlands) analysis was performed wherein the spectra was produced by the *θ*-2*θ* method [18,19]. The XRD was operated with 45 kV and 30 mA at room temperature. The anode material was Cu and the K-α (irradiation) was 1.544 Å. The scan range was 10°–90° and the step size was 0.167°. The intensities of the amorphous (2*θ* =18°) and crystal (2*θ* = 22°) regions, as reported by Segal *et al*. [22], were used to calculate CrI as follows:

$$\left(\mathrm{CrI}\right)=\frac{I_{22^{\circ }}-I_{18^{\circ }}}{I_{22^{\circ }}}\times 100\;\left(\%\right)$$

where *I*_*θ°*_ is intensity at the corresponding *θ*.

An FTIR spectroscopy analysis was also performed. The transmission the FTIR spectra were obtained using the FTIR spectrometer on an ambient atmosphere bench (Perkin-Elmer, Spectrum GX). The instrument was equipped with liquid-nitrogen-cooled mercury cadmium tellurium (MCT). The resolution of the spectra was 4 cm^-1^, and 256 scans were included to increase the signal-to-noise ratio [20,21].

## Abbreviations

CBU: Cellobiase unit; CCD: Central composite design; CrI: Crystallinity index; CV: Coefficient of variance; DAR: Dilute acid and aqueous ammonia combined pretreated rice straw; DNS: Dinitrosalicylic acid; DOE: Design of experiment; DR: Dilute-acid-pretreated rice straw; FPU: Filter paper unit; FTIR: Fourier transform infrared; HMF: Hydroxymethylfurfural; HPLC: High-performance liquid chromatography; MCT: Mercury cadmium tellurium; NREL: National Renewable Energy Laboratory; pNPG: *p*-nitrophenyl-*β*-D-glucopyranoside; R2: Determination coefficient; RR: Raw rice straw; RSM: Response surface methodology; SAA: Soaking in aqueous ammonia; XMG: Xylose, mannan and galactan; XRD: X-ray diffractometry.

## Competing interests

The authors declare that they have no competing interests.

## Authors’ contributions

SBK performed overall experiment containing statistical experiment design and wrote the manuscript. SJL, JHL and YRJ helped with the overall experiments of the pretreatment and instrumental analysis. LPT, YU and JSK advised on the fermentation process and preparation of media. CP and SWK coordinated the experimental design and reviewed the manuscript. All authors read and approved the final manuscript.
